# Mortality in prenatally detected congenital small bowel obstructions: systematic review and meta-analysis

**DOI:** 10.1016/j.xagr.2026.100619

**Published:** 2026-02-23

**Authors:** Hanna Heinrich, Ian Koorn, Nerissa P. Denswil, Elisabeth van Leeuwen, Ingeborg H. Linskens, Eva Pajkrt

**Affiliations:** 1 Department of Obstetrics and Gynecology (Heinrich, Koorn, Leeuwen and Pajkrt), Amsterdam UMC Location University of Amsterdam, Amsterdam, The Netherlands; 2 Amsterdam Reproduction and Development (Heinrich, Koorn, Leeuwen and Linskens), Amsterdam, The Netherlands; 3 Department of Research Support, Medical Library (Denswil), Amsterdam University Medical Centers, Amsterdam, The Netherlands; 4 Department of Obstetrics and Gynecology (Linskens), Amsterdam UMC Location Vrije Universiteit Amsterdam, Amsterdam, The Netherlands

**Keywords:** congenital small bowel obstructions, prenatal detection, intrauterine fetal demise, postnatal death

## Abstract

**Objective:**

To assess the risk of intrauterine fetal demise (IUFD) and postnatal death, and timing of demise in fetuses with a small bowel obstruction, in relation to isolated and nonisolated anomalies.

**Data sources:**

Embase (Ovid), MEDLINE (Ovid), and Cochrane Library were searched from inception to November 27, 2025.

**Study eligibility criteria:**

We excluded studies before the year 2000 to reduce the possible impact of improved prenatal and neonatal care on the outcome measures. Cohort studies and case series of >10 cases reporting on outcome (stillbirths and (post)neonatal death) of prenatally detected small bowel obstructions were included. Studies, including various types of gastrointestinal obstructions and both pre- and postnatally detected cases, were eligible if prenatally suspected congenital small bowel obstruction cases could be analyzed separately. No language restriction was applied. Cases solely reporting on the outcome on liveborn cases, animal studies, and conference abstracts were excluded.

**Methods:**

The primary outcome was IUFD. The secondary outcome was postnatal death. Data on associated structural and chromosomal anomalies were collected to evaluate the occurrence of mortality in isolated and nonisolated cases. Meta-analysis was performed to calculate the pooled proportions of IUFD and postnatal death, with separate analyses conducted for duodenal and jejunoileal obstructions.

**Results:**

The systematic review included 20 studies of 774 fetuses with small bowel obstruction. The pooled risk of IUFD was 6.4% [95% CI, 4.6%–8.9%]. Among IUFD cases with available information on additional anomalies, 43.8% (14/32) were classified as isolated. Median gestational age at IUFD was 33+3 weeks, IQR 32+4−34+2 weeks. Duodenal obstruction was associated with a pooled IUFD risk of 6.1% [95% CI 3.6%–10.0%] and jejunoileal obstruction with a pooled IUFD risk of 5.3% [95% CI, 2.6%–10.6%]. In addition, the pooled postnatal death risk was 8.5% [95% CI, 5.0%–14.2%], the majority of which occurred in nonisolated cases. The pooled postnatal death risk for suspected duodenal obstructions was 11.2% [95% CI, 6.3%–19.2%] and 3.9% [95% CI, 1.4%–10.4%] for jejunoileal obstructions.

**Conclusions:**

Our findings suggest that the risk of IUFD in fetuses with small bowel obstruction might not solely be attributable to additional structural or chromosomal anomalies. While evidence supporting the benefits of daily fetal monitoring on outcomes remains limited, monitoring from 32 to 33 weeks of gestation may be appropriate given the substantial risk of IUFD. Counseling should address both the absolute risks and the uncertain impact of monitoring. To mitigate the risk of term IUFD, induction of labor from 37 weeks could be considered while reducing the risks associated with premature delivery.

**AJOG Global Reports at a Glance**

**Why was this study conducted?**

Previous individual studies have reported on the risk of intrauterine fetal demise (IUFD) in fetuses with congenital small bowel obstruction. However, the limited sample sizes in prior studies preclude a more precise estimation of IUFD risk and the role of fetal monitoring has yet to be established. Improved insights in the risk of adverse outcome could enhance counseling of future parents and potentially also lead to fetal monitoring strategies.

**Key findings?**

This meta-analysis found a pooled risk of IUFD in fetuses with SBO of 6.4%, which also occurs in isolated cases, suggesting that the risk of IUFD might not solely be related to associated anomalies. The pooled risk of postnatal death is 8.5%.

**What does this add to what is known?**

Fetal monitoring from 32 to 33 weeks may be appropriate due to the timing and substantial risk of IUFD. Subsequently, induction from 37 weeks could be considered to mitigate the risk of term IUFD while minimizing the risks of prematurity in neonates likely requiring surgical intervention.

## Introduction

Congenital small bowel obstructions occur in 0.4–12.8 cases per 10,000 live births, with a general distinction made between duodenal obstructions and jejunoileal obstructions.^1^, ^2^, ^3^, ^4^^,^^5^ Especially, duodenal obstructions are associated with chromosomal abnormalities, in particular trisomy 21, and approximately 50% of cases exhibit additional structural anomalies.^6^

Previous studies have reported on the risk of intrauterine fetal demise (IUFD) in fetuses with congenital small bowel obstruction.^7^^,^^8^^,^^9^ In other congenital anomalies such as gastroschisis, a higher rate of IUFD has been described, prompting recommendations for fetal monitoring in the third trimester, while acknowledging delivery at term or near-term, rather than prematurely, is associated with improved perinatal outcomes.^10^, ^11^, ^12^, ^13^^,^^14^ However, for congenital small bowel obstructions, the limited sample sizes in prior studies preclude a more precise estimation of IUFD risk in fetuses with a congenital small bowel obstruction, and the role of daily fetal monitoring has yet to be established. Improved insights in the risk of adverse outcomes could enhance counseling of future parents and potentially also lead to fetal monitoring strategies.

Consequently, the aim of this systematic review is to evaluate the outcomes of fetuses with small bowel obstruction, focusing on the associated risk of fetal and neonatal mortality and timing of demise in relation to isolated and nonisolated anomalies. Additionally, this study will aim to provide recommendations regarding fetal monitoring and the timing of delivery.

## Objective

To evaluate the outcomes of fetuses diagnosed with small bowel obstruction, specifically examining the risk of fetal and neonatal mortality and the timing of demise in cases of isolated versus nonisolated duodenal and jejunoileal obstructions.

## Methods

This systematic review was performed according to the Preferred Reporting Items for Systematic Reviews and Meta-Analyses (PRISMA^15^). An a-priori-designed protocol was published in the PROSPERO international database for systematic reviews (CRD 42023428015).

### Data sources and search strategy

A medical information specialist (ND) performed a search in Embase (Ovid), MEDLINE (Ovid), and Cochrane Library from inception to November 27, 2025, to identify eligible studies (supplementary material). The search strategy used a combination of controlled terms (MeSH terms) and free text terms and synonyms for “intestinal atresia,” “small bowel obstruction,” “duodenal obstruction,” “jejunoileal obstruction” and “prenatal,” “perinatal” or “neonatal period.” No language and date restrictions were applied to the search. The bibliographic records retrieved were imported in Endnote and deduplicated using DeDupEndNote, and studies were screened and selected using Rayyan.^16^^,^^17^

### Eligibility criteria and study selection

The primary outcome measure was IUFD occurring after 20 weeks of gestation.^18^^,^^19^ Stillbirth, late miscarriage, and intrauterine demise were all classified as IUFD. The secondary outcome measure was postnatal death, defined as death after birth during the follow-up period of the selected articles. Neonatal death, defined as death within the first 28 days of life, was reported separately when possible.^20^

We included both retrospective and prospective cohort studies in cases with prenatally suspected congenital small bowel obstruction reporting on the primary outcome. Studies including various types of gastrointestinal obstructions and both pre- and postnatally detected cases were eligible if prenatally suspected congenital small bowel obstruction cases could be analyzed separately.

We excluded studies before the year 2000 to reduce the possible impact of improved prenatal and neonatal care on the outcome measures. In addition, we excluded case reports and case series of fewer than 10 cases, studies solely reporting on the postnatal outcome of liveborn cases with a congenital small bowel obstruction, animal studies, and conference abstracts. If manuscripts reported on the same population, the manuscript most relevant to our study was included.

### Data extraction

Two investigators independently reviewed all potentially eligible studies based on title and abstract (H.H. and I.K.). After title and abstract screening, the relevant full-text articles were retrieved and evaluated for eligibility by both investigators. Disagreement over eligibility was resolved by discussion between the 2 reviewers. The reference lists of the included articles were reviewed to identify any additional relevant studies (snowballing). Any persisting disagreement regarding eligibility was discussed with a fetal medicine specialist (EP).

Extracted data included study settings and population, baseline characteristics, prenatal diagnosis, gestational age at diagnosis, associated structural and chromosomal anomalies, adverse outcomes (termination of pregnancy, IUFD, neonatal death), timing of adverse outcomes, prenatal fetal monitoring strategies, intrapartum management, live birth rates, survival rates, and frequency of surgical intervention. If not all required data were available, the authors were approached to complete the required data.

### Validity assessment

The risk of bias of all included articles was assessed using the Quality in Prognosis Studies (QUIPS) tool for prognostic studies by 2 authors independently (H.H. and I.K.). Emphasis was placed on the quality of reporting regarding confounders for IUFD, such as associated anomalies, fetal monitoring strategies, and interventions that reduce the number of fetuses at risk (eg, termination of pregnancy and iatrogenic preterm birth), as well as on the detail in reporting of IUFD cases themselves. Discrepancies were resolved by consensus, with consultation of a senior reviewer (EP) when necessary.

### Data synthesis

We performed a meta-analysis for the primary and secondary outcomes of IUFD and postnatal death. To evaluate IUFD and postnatal death rates within the studies, cases with unknown pregnancy outcomes were excluded from the analysis, as were cases in which the pregnancy was terminated, as these cases were no longer at risk of adverse outcome. A subgroup analysis was performed on the level of intestinal obstruction (duodenal/jejunoileal) as previous literature has a higher rate of associated structural and chromosomal anomalies in duodenal obstructions. Cases labeled as jejunoileal obstruction, small bowel obstruction (excluding duodenal obstructions), jejunal obstruction, ileal obstruction, or midgut volvulus were classified under jejunoileal obstruction. Studies that did not specify the anatomical level of small bowel obstruction were excluded from the subgroup analysis.

We performed a one-arm meta-analysis for proportions using a generalized linear mixed model with a logit transformation, as our outcome measures are binary and from single-arm studies with relatively small sample sizes.^21^^,^^22^ To calculate prediction intervals, the *t*-distribution was used. The *I*² and *τ*² statistics were used to assess between-study heterogeneity.^23^^,^^24^ The Hartung and Knapp method was used to adjust test statistics and confidence intervals. Publication bias was assessed using a Doi plot and the LFK index, as these are considered more robust than funnel plots in prevalence studies.^25^^,^^26^ Statistical analyses and generation of forest plots were carried out using RStudio (RStudio PBC^27^). *P* values <.05 were considered statistically significant.

## Results

### Study selection and risk of bias

**Figure 1:**
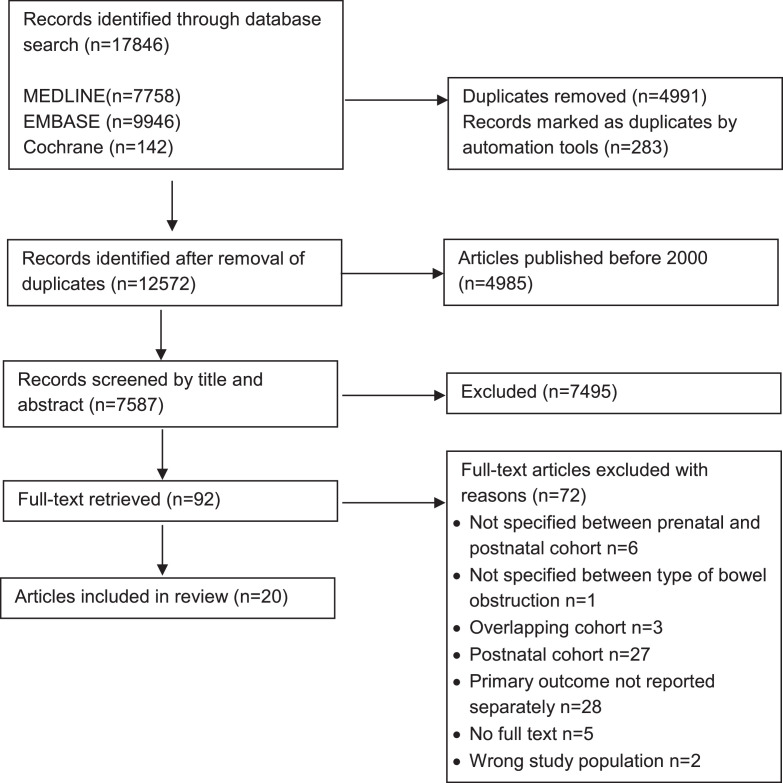
Flowchart of selection.

A total of 7587 articles were identified after removing duplicates and applying a publication year filter. titles, and abstracts were screened, resulting in 92 articles selected for full-text review. Of these, 20 studies were included in the systematic review and meta-analysis (Figure 1). Following the QUIPS tool assessment (Appendix A), high risk of bias was found in 3 studies. Limitations of studies included minimal or no reporting on fetal monitoring and intrapartum management, as well as limited reporting on associated chromosomal and structural anomalies, which may have affected the risk of IUFD.

Heinrich. Mortality in prenatally detected congenital small bowel obstructions: systematic review and meta-analysis. Am J Obstet Gynecol MFM 2026.

### Study characteristics

The general characteristics of the individual studies are provided in Supplementary Table 1. The 20 included studies reported on 774 cases with individual sample sizes varying between 13 and 111 cases. Among the selected studies, 10 studies reported only on suspected duodenal obstructions,^7^^,^^28^, ^29^, ^30^, ^31^, ^32^, ^33^, ^34^, ^35^^,^^36^ 3 reported only on suspected jejunoileal obstructions,^37^^,^^38^^,^^39^ 6 studies distinguished between duodenal and jejunoileal obstructions,^8^^,^^9^^,^^40^, ^41^, ^42^^,^^43^ and 1 study did not distinguish between the levels of the small bowel obstruction.^44^ A specific prenatally suspected level of obstruction suitable for subgroup analysis was available in 555 cases of duodenal obstruction and in 206 cases of jejunoileal obstruction.

Seventeen studies reported on chromosomal anomalies in cases with prenatally suspected small bowel obstruction, with a chromosomal anomaly identified in 19.8% (140/707) (Supplementary Table 2).^7^^,^^8^^,^^9^^,^^28^^,^^30^, ^31^, ^32^, ^33^, ^34^^,^^35^^,^^37^^,^^38^^,^^40^^,^^41^^,^ ^42^^,^^44^^,^^45^

Thirteen studies reported specifically on chromosomal anomalies in cases with suspected duodenal obstruction, which identified a chromosomal anomaly in 24.3% (126/519).^7^^,^^8^^,^^9^^,^^28^^,^^30^^,^^31^^,^^32^^,^^34^^,^^35^^,^^40^^,^^41^^,^^42^^,^^45^ Of the 7 studies that reported on chromosomal anomalies in jejunoileal obstructions, this occurred in 6.3% (12/175).^8^^,^^9^^,^^37^^,^^38^^,^^40^^,^^41^^,^^42^ Termination of pregnancy was performed in 99/774 (12.8%) cases, ranging from a termination rate of 0 to 75% among studies. The highest rate of pregnancy terminations was reported by Yin et al. in which 75% (30/40) of cases were terminated. In contrast, in 6 studies no terminations of pregnancy were reported.^8^^,^^33^^,^^38^^,^^39^^,^^42^^,^^45^ By subtracting the number of pregnancy terminations (*n*=99) and cases lost to follow-up (*n*=7), 668 cases were at risk of adverse outcome.

### Synthesis of results

#### Intrauterine fetal demise

**Figure 2:**
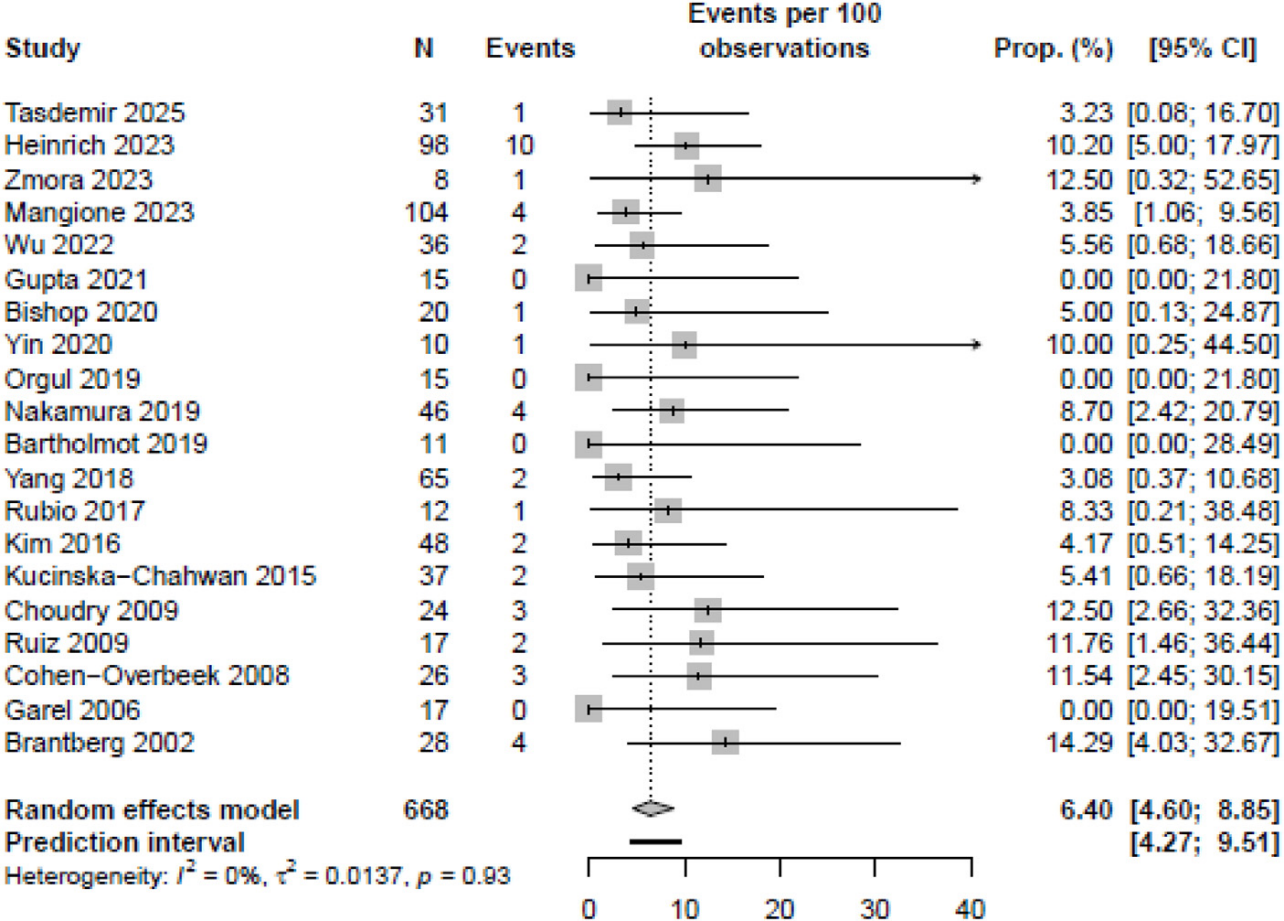
Forest plot illustrating the (pooled) proportions and prediction intervals of intrauterine fetal death (IUFD) prevalence among fetuses with a small bowel obstruction.

The meta-analysis showed a pooled risk of IUFD of 6.4% [95% CI, 4.6%–8.9%] (Figure 2). Gestational age at IUFD was reported in 9 of the studies, ranging between 26 and 38 weeks of gestation (median 33+3 weeks, IQR 32+4 −34+2 weeks).^7^^,^^8^^,^^9^^,^^31^^,^^33^^,^^34^^,^^35^^,^^38^^,^^44^ The characteristics of IUFD cases were documented in 11/20 studies (Supplementary Table 3).^7^^,^^8^^,^^9^^,^^30^^,^^31^^,^^33^^,^^34^^,^^35^^,^^38^^,^^41^^,^^44^ Of these cases, 43.8% (14/32) were classified as isolated. In the isolated cases, IUFD occurred between 30+6 and 37+2 weeks, whereas in nonisolated cases, IUFD occurred between 26+0 and 38+0 weeks.^7^^,^^8^^,^^9^^,^^31^^,^^33^^,^^34^^,^^35^^,^^38^^,^^44^ Four studies with a limited sample size (*n* = 11, 15, 15, and 17) reported no cases of IUFD.^37^^,^^42^^,^^43^^,^^45^ The pooled risk of IUFD for the subgroup of suspected duodenal obstructions was 6.1% [95% CI, 3.6%–10.0%], whereas the pooled risk for IUFD in suspected jejunoileal obstructions was 5.3% [95% CI, 2.6%–10.6%] (Appendix B and C). Live birth rates varied from 87.5% to 100% between studies (Supplementary Table 2). The LFK indices for all IUFD cases, as well as for IUFD associated with duodenal and jejunoileal obstructions, were −4.03, −4.44, and −1.28, respectively, indicating asymmetry and potential biases in overall IUFD cases and in those related to duodenal obstruction (Supplementary Material).

Heinrich. Mortality in prenatally detected congenital small bowel obstructions: systematic review and meta-analysis. Am J Obstet Gynecol MFM 2026.

#### Postnatal death

**Figure 3:**
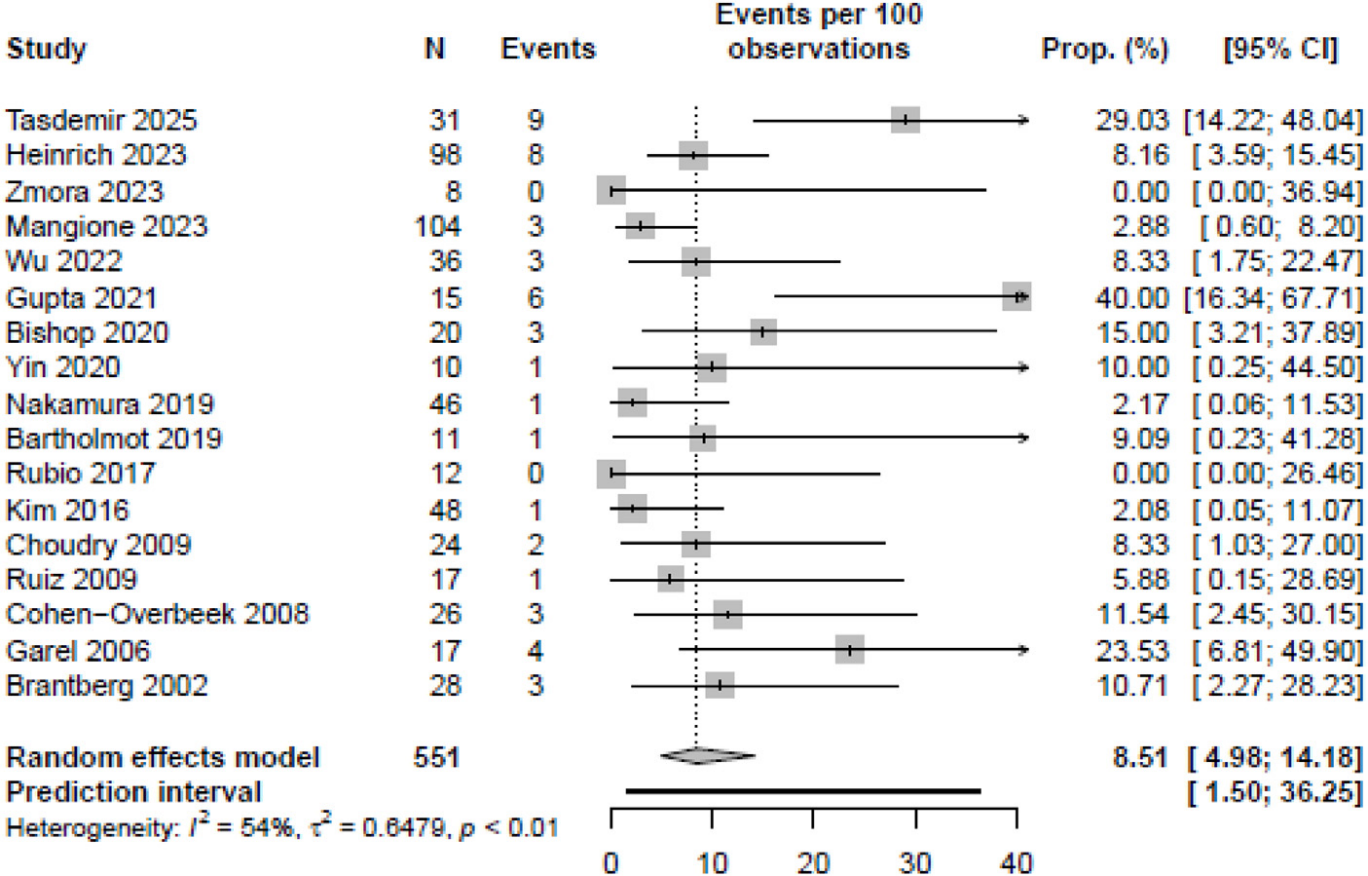
Forest plot illustrating the (pooled) proportions and prediction intervals of neonatal death (NND) prevalence among fetuses with a small bowel obstruction.

Seventeen studies reported on postnatal death.^7^^,^^8^^,^^9^^,^^28^, ^29^, ^30^, ^31^, ^32^, ^33^, ^34^^,^^35^^,^^37^, ^38^, ^39^, ^40^^,^^41^^,^^43^^,^^44^^,^^45^ The meta-analysis demonstrated a pooled risk of neonatal death of 8.5% (95% CI, 5.0%–14.2%) (Figure 3). Two studies specifically reported no cases of postnatal death within their cohort.^38^^,^^44^ For the cases with a prenatally suspected duodenal obstruction, the pooled risk of postnatal death was 11.2% [95% CI, 6.3%–19.2%] (Appendix D). The pooled risk of postnatal death in cases with suspected jejunoileal obstruction was 3.9% [95% CI, 1.4%–10.4%] (Appendix E). Thirteen studies reported on associated anomalies in cases of postnatal death, 9 of which only found postnatal death in nonisolated cases.^7^^,^^8^^,^^9^^,^^29^, ^30^, ^31^^,^^34^^,^^40^^,^^41^ However, postnatal death was reported in 8 (8/44) isolated cases.^33^^,^^35^^,^^40^^,^^45^ The majority of the postnatal deaths occurred in the neonatal period, although instances were reported up to 2.5 years of age.^35^ NND (<28 days of age) was specifically mentioned in 10 studies, accounting for 22/27 cases of postnatal death.^7^^,^^8^^,^^9^^,^^29^^,^^31^^,^^33^^,^^34^^,^^37^^,^^40^^,^^43^ Overall survival varied between studies from 60.0% to 93.8%. The LFK indices for NND and NND associated with duodenal and jejunoileal obstructions were −1.36, −0.95, and −0.65, indicating no to minor asymmetry (Supplementary Material).

Heinrich. Mortality in prenatally detected congenital small bowel obstructions: systematic review and meta-analysis. Am J Obstet Gynecol MFM 2026.

## Comment

### Principle findings

This systematic review and meta-analysis found a pooled IUFD risk of 6.4% in fetuses with prenatally suspected small bowel obstruction. Among IUFD cases with available additional information, 43.8% were classified as isolated. The pooled postnatal death risk was 8.5%, the majority of which occurred in nonisolated cases.

### Strengths and limitations

The strengths of this review are the inclusion of all relevant studies on pregnancy outcomes in prenatally diagnosed small bowel obstructions, with separate analyses of IUFD and postnatal mortality. The meta-analysis followed standardized PRISMA methodology, with a registered protocol in the PROSPERO database and quality assessment of the included studies.

However, several limitations should be recognized. First, heterogeneity in documentation of concomitant anomalies prevented a separate meta-analysis of IUFD risk in isolated SBO cases. As studies reported structural and chromosomal anomalies separately (without reporting potential cases with both) and also classified cases without genetic testing as isolated, the true number of isolated studies could not be determined. Due to this heterogeneity in documentation, we chose to present concomitant anomalies in IUFD cases descriptively. Potential underestimation of the number of isolated cases in the denominator could have subsequently inflated the IUFD risk in this isolated SBO group. Thus, while our pooled IUFD rate likely overestimates the true IUFD rate for isolated cases, this would likely not be prevented by performing a separate analysis for isolated cases. Nonetheless, as almost half of the IUFD cases were classified as isolated cases, this may suggest that associated anomalies do not solely contribute to the risk of adverse outcome. However, although the current evidence is suggestive, it is insufficient to establish a definitive mechanism. Future studies should focus on consistent documentation of isolated and nonisolated cases to better assess the risk of adverse outcomes in isolated cases.

Second, there may be a selection bias in the included cases. Small bowel obstructions are often detected in the late second- or third trimester due to increased gastric emptying and fetal swallowing movements. Additionally, selective reporting or underreporting of negative outcomes may further contribute to bias. In countries where third-trimester ultrasound is not routinely performed, prenatal detection rates are expected to be lower. Therefore, more severe cases with associated anomalies that receive follow-up or those with polyhydramnios prompting detection may be overrepresented in published studies, potentially overestimating the risk of mortality. However, in the absence of a standardized third trimester ultrasound, the reported outcomes offer a reasonable reflection on the expected prognosis in prenatally detected cases. When evaluating the Doi plots and LFK curves, major asymmetry was detected in overall IUFD (LFK −4.03) and duodenal IUFD (LFK −4.44). Although a positive publication bias was expected a priori, the observed negative LFK index does not reflect this, as smaller studies also reported low incidences of IUFD. This asymmetry may therefore be due to other forms of bias and should be considered when interpreting the results.^25^

Finally, methodological heterogeneity may have influenced the reported outcomes, particularly in handling false positives and loss to follow-up. Some studies excluded false positives from further analysis, whereas others included them. Exclusion of false positives may impact risk estimates, as they are not yet identified prenatally, whereas their inclusion may lead to underestimation of IUFD risk in confirmed intestinal obstruction cases. Furthermore, autopsy was not performed in all cases of IUFD, thereby precluding definitive confirmation of small bowel obstruction. In this review, the handling of false positives, whether included or excluded, was evenly balanced across the studies. Similarly, excluding cases lost to follow-up or cases ending in pregnancy termination also has implications on the calculated outcomes. The wide range of pregnancy termination rates across studies (range between 0% and 75%) may reflect differences in societal norms and parental choices. Excluding these cases decreases the denominator of the cases at risk, which impacts the generalizability of this study. However, as it cannot be ascertained whether these cases were at risk, their exclusion yields a more accurate representation of the data. These methodological differences should be considered when interpreting results and should be addressed during counseling.

### Comparison with existing literature

Several theories have been proposed to explain the pathophysiological mechanisms of IUFD in cases of small bowel obstruction. Associated structural and chromosomal anomalies, particularly trisomy 21, are most frequently proposed as the underlying cause of mortality.^46^ However, this review highlights IUFD in apparently isolated cases of small bowel obstruction, suggesting possible additional mechanisms contributing to mortality. A proposed explanation is the development of umbilical cord ulcers due to reflux vomiting, where elevated bile acids and pancreatic enzymes in the amniotic fluid may damage Wharton’s jelly, exposing and disrupting umbilical vessels.^8^^,^^47^^,^^48^ Another hypothesis suggests vagal overactivity caused by distention of the upper gastrointestinal tract leading to bradycardia and asystole.^7^ Lastly, it is hypothesized that the aspiration of intestinal contents may lead to the uptake of bile acids in the fetal bloodstream, where elevated levels of bile acid could induce a fetal arrhythmia—a mechanism also proposed to explain IUFD in cases with maternal intrahepatic cholestasis of pregnancy.^49^

In order to mitigate the risk of IUFD in cases with small bowel obstruction, fetal monitoring could be considered as a management strategy. To date, there is not sufficient evidence that fetal monitoring will improve fetal outcome. Brantberg et al. reported on 4 cases of IUFD where, despite fetal monitoring, only 1 case presented because of reduced fetal movements with abnormal findings during ultrasound examination (bradycardia and poor cardiac contractility).^7^ In the remaining 3 cases, a normal fetal heart rate or CTG was documented on the same day or the day preceding the IUFD. However, a considerable proportion of the studies did not elaborate on monitoring strategies, for which it remains uncertain how and if this has impacted mortality risk. Given the substantial risk of IUFD and the median gestational age of IUFD at 33+3 weeks (IQR 32+4−34+5 weeks), initiating fetal monitoring from 32 to 33 weeks could be considered. Future parents should be informed of the risk of fetal demise and the importance of monitoring fetal movements while also recognizing the uncertain benefits and limitations of fetal monitoring in this context, as its efficacy in improving perinatal outcomes has not yet been validated.

Another possible solution to the prevention of sudden IUFD in fetuses is iatrogenic premature delivery. However, this approach requires careful evaluation of the risks of premature delivery in combination with the additional risks of surgery on a preterm neonate weighed against the risk of sudden IUFD.^50^ The incidence of term IUFD in this review was relatively low; however, included studies reported a median gestational age at delivery of approximately 36 weeks. This may reflect either an increased risk of preterm birth or a trend toward earlier induction of labor. Therefore, induction of labor from 37 weeks of gestation may be considered to mitigate the possible risk of late IUFD while reducing the risks associated with premature delivery. However, these recommendations are expert-opinion-based and remain preliminary, as prospective comparative studies or randomized trials are required to validate their efficacy before formal guideline adoption. To improve comparability and reproducibility, future studies should adopt standardized reporting. A minimal dataset should distinguish isolated from nonisolated cases, include details of genetic testing, pregnancy outcomes and outline prenatal monitoring strategies, and report postnatal or postmortem confirmation of SBO.

## Conclusions and implications

This systematic review and meta-analysis found a pooled risk of IUFD in fetuses with prenatally suspected small bowel obstruction of 6.4%. IUFD was also observed in isolated cases, suggesting that factors beyond associated anomalies contribute to adverse outcomes. While evidence supporting the benefits of fetal monitoring on outcomes remains limited, its use from 32 to 33 weeks of gestation onward may be appropriate given the substantial risk of IUFD. Parents should be informed of both this risk and the uncertain value of monitoring. To mitigate the risk of late IUFD, induction of labor from 37 weeks could be considered.

## CRediT authorship contribution statement

**Hanna Heinrich:** Writing – review & editing, Writing – original draft, Visualization, Validation, Software, Methodology, Investigation, Formal analysis, Data curation, Conceptualization. **Ian Koorn:** Writing – review & editing, Visualization, Software, Methodology, Formal analysis, Data curation. **Nerissa P. Denswil:** Writing – review & editing, Data curation. **Elisabeth van Leeuwen:** Writing – review & editing, Supervision, Conceptualization. **Ingeborg H. Linskens:** Writing – review & editing, Supervision. **Eva Pajkrt:** Writing – review & editing, Supervision, Conceptualization.

## Appendix Supplementary materials

Image, application 1

Image, application 2

Image, application 3

Image, application 4

Image, application 5

Image, application 6

Image, application 7

## Footnotes

Supplementary material associated with this article can be found in the online version at doi:10.1016/j.xagr.2026.100619.
