# Supplementary material for: Mortality in prenatally detected congenital small bowel obstructions: systematic review and meta-analysis
Source: AJOG Glob Rep. 2026 Feb 23;6(2):100619. doi: 10.1016/j.xagr.2026.100619 (PMC13019568; doi:10.1016/j.xagr.2026.100619)

**DOI Plot 1.** Intrauterine fetal demise (IUFD) with small bowel obstruction


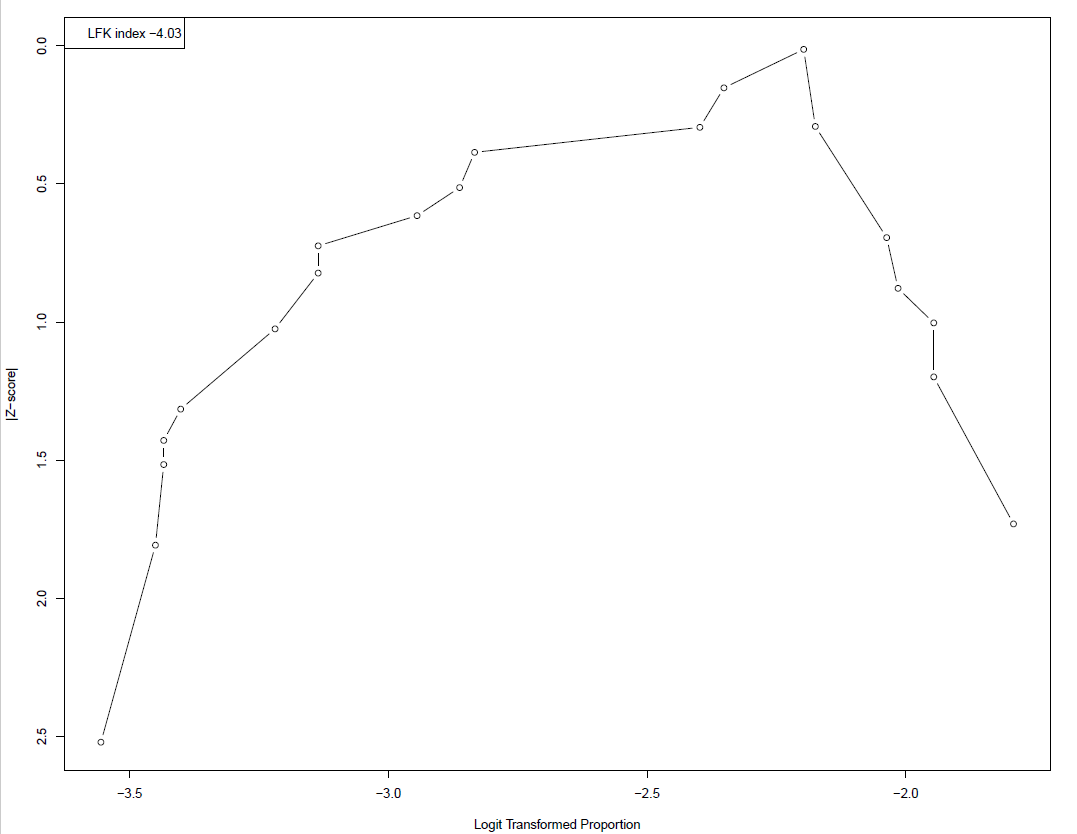


**DOI Plot 2**. Neonatal death (NND) with small bowel obstruction


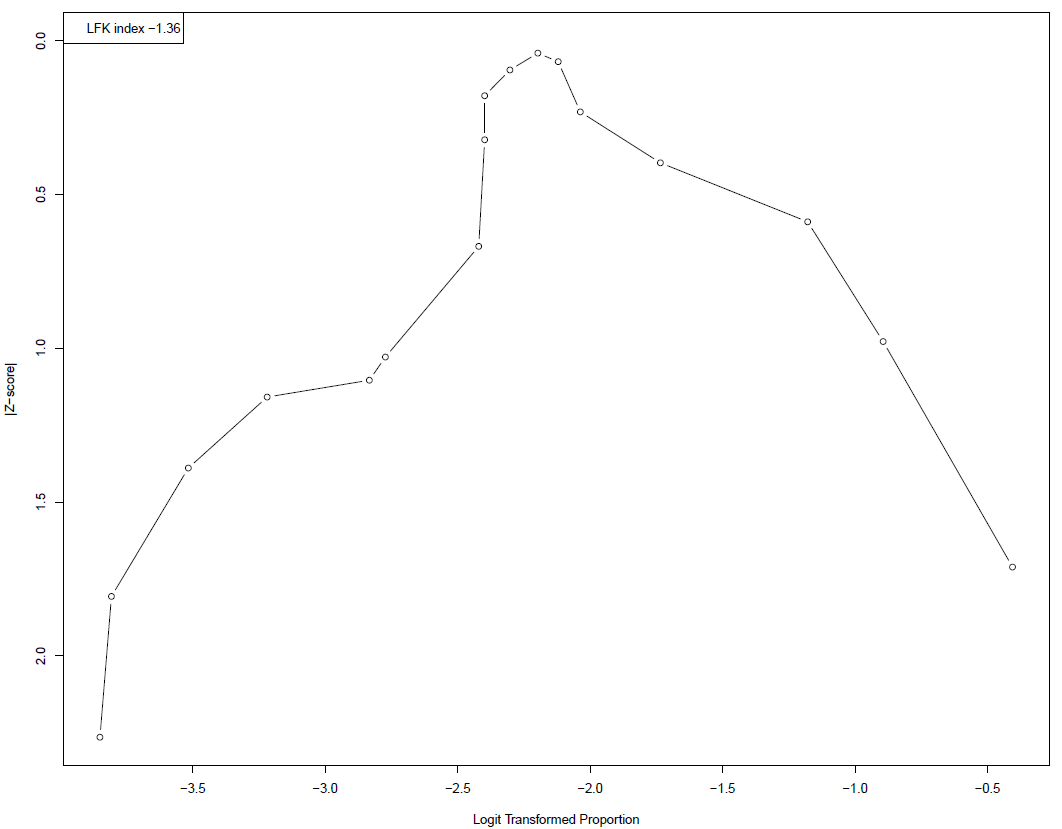


**DOI Plot 3.** Intrauterine fetal demise (IUFD) in cases with duodenal obstruction


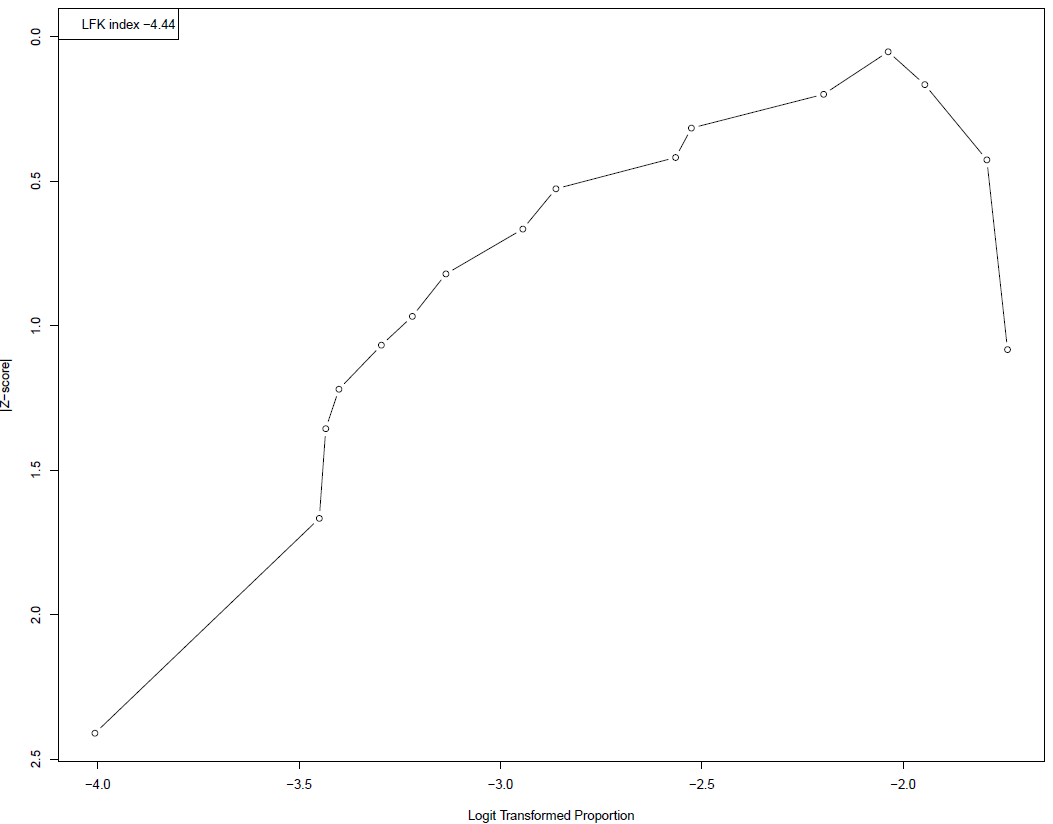


**DOI Plot 4.** Intrauterine fetal demise (IUFD) in cases with jejunoileal obstruction
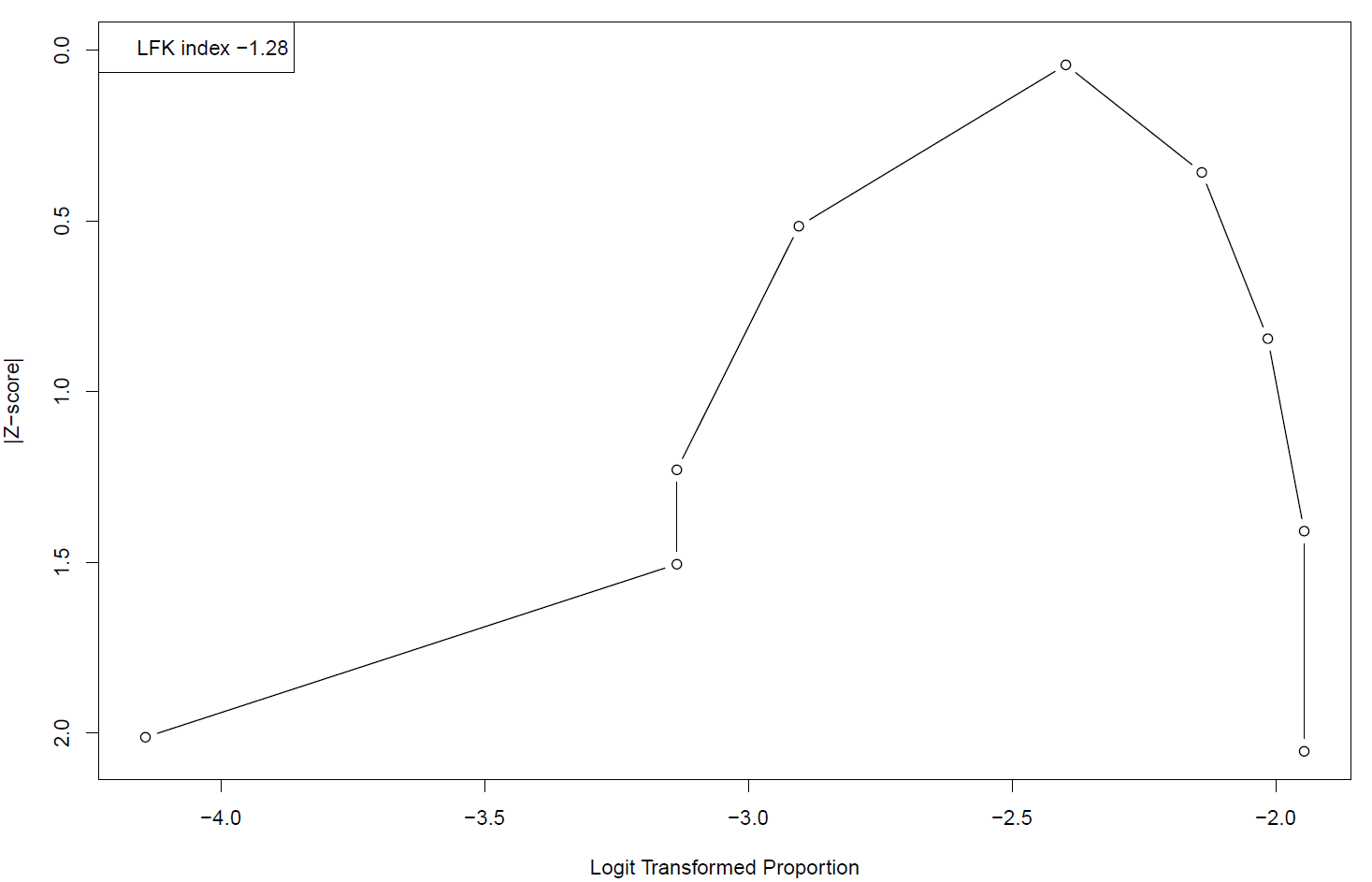


**DOI Plot 5.** Neonatal death (NND) in cases with duodenal obstruction


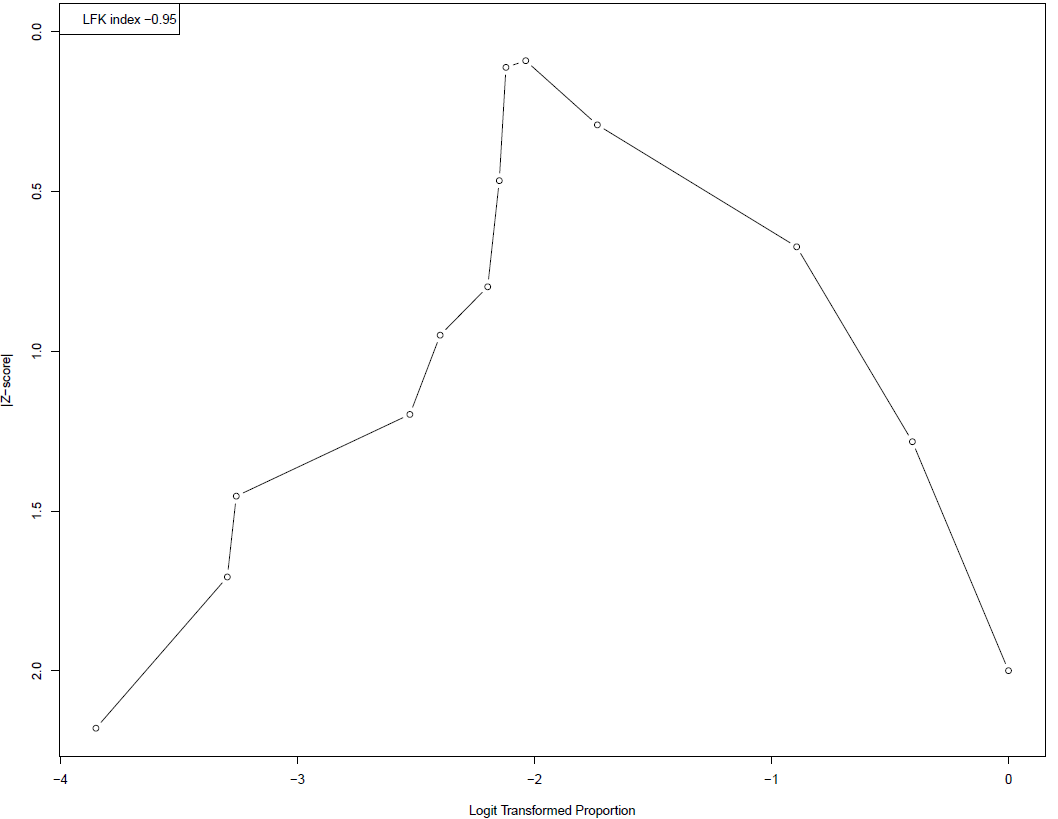


**DOI Plot 6.** Neonatal death (NND) in cases with jejunoileal obstruction
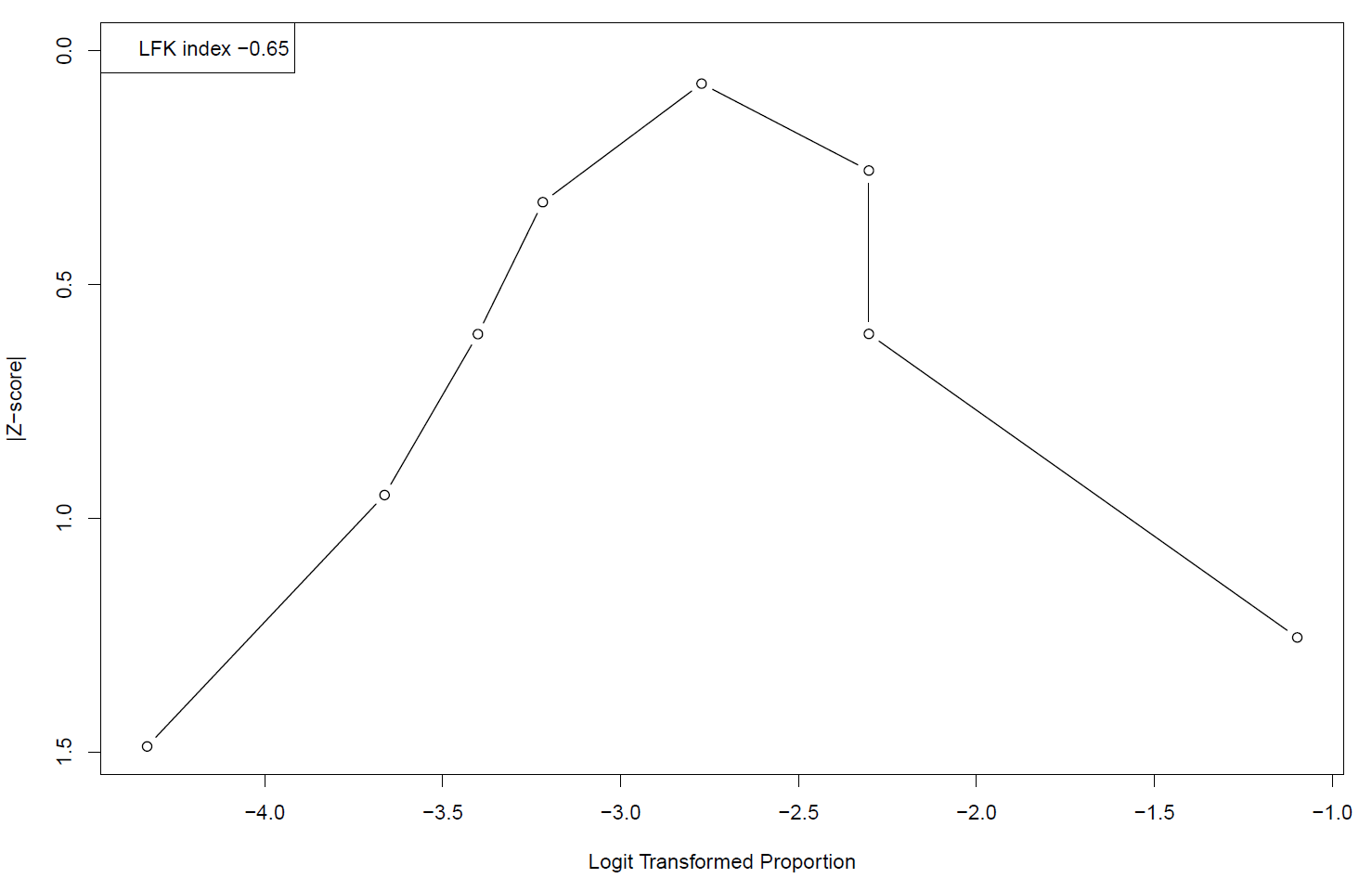

Supplement: Supplementary file 1 [file mmc1.docx]
