# Supplementary material for: Mortality in prenatally detected congenital small bowel obstructions: systematic review and meta-analysis
Source: AJOG Glob Rep. 2026 Feb 23;6(2):100619. doi: 10.1016/j.xagr.2026.100619 (PMC13019568; doi:10.1016/j.xagr.2026.100619)
Supplement: Supplementary file 2 [file mmc2.docx]

Table 1: Characteristics of included studies and monitoring strategies

| **Author (year)** | **Country** | **Study period/** | **Study design** | **Prenatal cases,** *n* | **Fetal monitoring strategies** | **Intrapartum management** |
| --- | --- | --- | --- | --- | --- | --- |
| Taşdemir (2025)^37^ | Turkey | 2018-2024/ | Retrospective,  FP excluded | DO 34 | Weekly or biweekly ultrasound evaluations. CTG + ultrasound after 32 weeks. | No maximum gestational week limit regarding timing of delivery, no special obstetric indication to perform caesarean section. |
| Heinrich (2023)^9^ | Netherlands | 2007-2021/ | Retrospective,  FP excluded | DO 76  JIO 31 | Weekly or biweekly ultrasound evaluations. | No strict upper limit for timing of delivery, however over the years more often agreed upon induction of labor after 37 weeks of gestation. Mode of delivery was not determined by suspicion of SBO. |
| Zmora (2023)^46^ | Israel | 2007-2021 | Retrospective,  All cases included | SBO 13 | - | - |
| Mangione (2023) ^43^ | France | 2016-2018 | Prospective,  All cases included | DO 30  JIO 81 | - | - |
| Wu (2022)^42^ | China | 2012-2021/ | Not specified,  All cases included | DO 43  JIO 16 | - | - |
| Gupta (2021)^47^ | India | 2016-2019 | Prospective,  All cases included | DO 15 | - | None of the malformations was an indication for  cesarean section. |
| Bishop (2020) ^32^ | USA | 2008-2017/ | Retrospective,  FP excluded | DO 21 | - | - |
| Yin (2020) ^33^ | China | 2016-2019/ | Retrospective,  All cases included | DO 40 | - | - |
| Orgul (2019) ^44^ | Turkey | 2005-2015 | Retrospective,  FP excluded | DO 12  JIO 3 | - | - |
| Nakamura (2019)^8^ | Japan | 2003-2017 | Retrospective,  FP excluded | DO 27  JIO 19 | From 2010 weekly US and electric fetal monitoring every 2 days after 34 weeks. | From 2010 onwards caesarean section at 36 weeks of gestation. Other indications for delivery were decided by the attending obstetricians. |
| Bartholmot (2019)^39^ | France | 2006-2017 | Retrospective,  All cases included | JIO 13 | Weekly ultrasound examination and fetal heart rate monitoring | Delivery not based on strict intestinal dilatation cutoff but on a bundle clinical and sonographic arguments according to GA. |
| Yang (2018)^34^ | China | 2011-2015/ | Retrospective,  All cases included | DO 71 | - | - |
| Rubio (2017)^40^ | USA | 2005-2015/ | Retrospective,  All cases included | JIO 12 | - | - |
| Kim (2016)^35^ | Korea | 1995-2014/ | Retrospective,  FP excluded | DO 51 | - | Caesarean section according to other obstetric indications such as breech or previous caesarean are present/ labor induction according to each clinicians' clinical decision |
| Kucinska-Chahwan (2015)^18^ | Poland | 1998-2013/ | Retrospective,  All cases included | DO 42 | - | - |
| Choudhry (2009)^19^ | UK | 1995-2004/ | Retrospective,  FP excluded | DO 29 | - | - |
| Ruiz (2009)^41^ | USA | 2003-2006/ | Retrospective,  All cases included | JIO 20 | - | - |
| Cohen-Overbeek (2008)^36^ | Netherlands | 1991-2003/ | Retrospective,  FP excluded | DO 28 | Regular standard US monitoring, no extra CTG monitoring unless symptoms during regular observations | Spontaneous deliveries were awaited. Vaginal delivery unless obstetric reasons dictated otherwise. |
| Garel (2006)^45^ | France | 2000-2005/ | Prospective,  FP excluded | DO 7  JIO 11 | - | - |
| Brantberg (2002)^7^ | Norway | 1985-2000/ | Prospective,  FP excluded | DO 29 | Not specifically mentioned. In cases of IUFD 3/4 cases were admitted for CTG monitoring. | - |

Only first author is given for each study. DO= Duodenal obstruction, JIO= Jejunoileal obstruction, SBO= Small bowel obstruction, FP=False positives

Table 2: Clinical characteristics and outcomes of 759 cases with a small bowel obstruction

| **Author** | **Prenatal cases**  *n* | **Prenatal Associated anomalies**  *n (%)* | **Chromosomal /genetic anomalies**  *n (%)* | **Type of chromosomal/ genetic anomaly** | **Lost to FU** **/ TOP**  *n/n* | **Cases**  **at risk**  *n* | **IUFD^*^**  *n (%)* | **Livebirths^*^**  *n (%)* | **GA delivery**  median [IQR] or (range) | **Postnatal death**  *n (%)* | **Survival^*^**  *n (%)* |
| --- | --- | --- | --- | --- | --- | --- | --- | --- | --- | --- | --- |
| Taşdemir^37^ | DO 34 | DO 9 (26.5) | DO 19 (55.9)^&^ | DO : Trisomy 21 15  Deletion 7q11.23 1  46,XX,(der 21) 1  47,XX,inv(9)(p11q13),+2 1  Triploidy 1 | 0 / 3 | 31 | 1 (3.2) | 30 (96.8) | DO 36.0 [35.0-38.25] | 9 (29.0) | 21 (67.7) |
| Heinrich^9^, | DO 76  JIO 31 | DO 33 (43.4)  JIO 13 (41.9) | DO 22 (28.9)^&^  JIO 1 (3.2) | DO: Trisomy 21 21  HDAC8 mutation 1  JIO: ARID2 mutation 1 | 0 / 9 | 98 | 10 (10.2) | 88 (89.8) | DO 36.9 [34.6–37.9]  JIO 36.2 [34.1-37.3] | 8 (8.2) | 80 (81.6) |
| Zmora^46^ | SBO 13 | - | SBO 3 (23.1) | SBO: Trisomy 21 2  Jeune syndrome 1 | 0 / 5 | 8 | 1 (12.5) | 6 (75.0) | - | 0 | 6 (75.0) |
| Mangione^43^ | DO 30  JIO 81 | DO 20 (66.7)  JIO 22 (27.2) | DO 5 (16.7)^#^  JIO 5 (6.2) | DO: Trisomy 21 5  JIO: Cystic fibrosis 4  46XY,der(13)t(2; 13) 1 | 0 / 7 | 104 | 4 (3.8) | 100 (96.2) | - | 3 (2.9) | 97 (93.4) |
| Wu^42^ | DO 43  JIO 16 | DO 19 (44.2)  JIO 9 (43.7) | DO 11 (25.6)^#^  JIO 0 (0.0) | DO: Trisomy 21 10  17q12 duplication 1 syndrome | 4 / 19 | 36 | 2 (5.6) | 34 (94.4) | - | 3 (8.3) | 31 (86.1) |
| Gupta^47^ | DO 15 | DO 4 (26.7) | DO 2 (13.3) | DO: Trisomy 21 2 | 0/0 | 15 | 0 (0.0) | 15 (100.0) | - | 6 (40.0) | 9 (60.0) |
| Bishop^32^ | DO 21 | DO 8 (39.1) | DO 8 (38.1)^&^ | DO: Trisomy 21 6  Variant in ZIC3 gene 1 Microdeletion 4q22.3 1 | 0 / 1 | 20 | 1 (5.0) | 19 (95.0) | DO 35.6 (26.1-41.2) | 3 (15.0) | 16 (80.0) |
| Yin^33^ | DO 40 | DO 23 (57.5) | DO 0 (0.0)^&^ |  | 0 / 30 | 10 | 1 (10.0) | 9 (90.0) | DO 37.6 +/- 1.4 | 1 (10.0) | 8 (80.0) |
| Orgul^44^ | DO 12  JIO 3 | DO 1 (8.3)  JIO 1 (33.3) | DO 2 (16.7)^&^  JIO 0 (0.0) | DO: Trisomy 21 2 | 0 / 0 | 15 | 0 (0.0) | 15 (100.0) | -^$^ | -^$^ | -^$^ |
| Nakamura^8^ | DO 27  JIO 19 | DO 9 (33.3)  JIO 3 (15.8) | DO 10 (37.0)^&^  JIO 0 (0.0) | DO: Trisomy 21 9  46,XX, del(13)(q22q31) 1 | 0 / 0 | 46 | 4 (8.7) | 42 (91.3) | DO 36.3 (26.3-38.9)  JIO 36.3 (33.4-39.4) | 1 (2.2) | 41 (89.1) |
| Bartholmot^39^ | JIO 13 | - | JIO 2 (15.4)^#^ | JIO: Cystic fibrosis 2 | 0 / 2 | 11 | 0 (0.0) | 11 (100.0) | JIO Mean 36.6 (SD 2.1) | 1 (9.1) | 10 (90.9) |
| Yang^34^ | DO 71 | DO 8 (11.2) | DO 7 (9.9)^#^ | DO: Trisomy 21 7 | 0 / 6 | 65 | 2 (3.1) | 63 (96.9) | DO 36 (30.0-40.0) | - | - |
| Rubio^40^ | JIO 12 | - | JIO 3 (25.0)^&^ | JIO: Cystic fibrosis 3 | 0 / 0 | 12 | 1 (8.3) | 11 (91.7) | JIO 34.4 (26.0-Term) | 0 (0.0) | 11 (91.7) |
| Kim^35^ | DO 51 |  | DO 6 (11.7)^#^ | - | 3 / 0 | 48 | 2 (4.2) | 46 (95.8) | DO 37+0 [34+1-38+4] | 1 (2.1) | 45 (93.8) |
| Kucinska-Chahwan^18^ | DO 42 | DO 24 (57.1) | DO 21 (50.0)^#^ | DO: Trisomy 21 20  Triploidy 1 | 0 / 5 | 37 | 2 (5.4) | 35 (94.6) | DO 36.7 (28-41) | - | - |
| Choudhry^19^ | DO 29 | DO 21 (72.4) | - | - | 0 / 5 | 24 | 3 (12.5) | 21 (87.5) | - | 2 (8.3) | 19 (79.2) |
| Ruiz^41^ | JIO 20 | - | - | - | 0 / 3 | 17 | 2 (11.8) | 15 (88.2) | JIO Mean 37.1 weeks | 1 (5.9) | 14 (82.4) |
| Cohen-Overbeek^36^ | DO 28 | DO 17 (60.7) | DO 7 (25.0)^#^ | DO: Trisomy 21 7 | 0 / 2 | 26 | 3 (11.5) | 23 (88.5) | DO Isolated 35.4 (30.1-39.3)  DO Non-isolated 35.7 (29.3-39.1) | 3 (11.5) | 20 (76.9) |
| Garel^45^ | DO 7  JIO 11 | DO 1 (14.3)  JIO 4 (36.4) | - | - | 0 / 1 | 17 | 0 (0.0) | 17 (100.0) | - | 4 (23.5) | 13 (76.5) |
| Brantberg^7^ | DO 29 | DO 15 (51.7) | DO 6 (20.7)^#^ | DO: Trisomy 21 6 | 0 / 1 | 28 | 4 (13.8) | 24 (85.7) | DO 35+5 (29+1 - 39+3) | 3 (10.3) | 21 (75.0) |

Only first author is given for each study. ^*^Calculated as percentage of cases at risk. ^#^Based on prenatal genetic testing. ^&^Based on both prenatal- and postnatal genetic testing. . ^$^Not reported for prenatal cohort separately. DO= Duodenal obstruction, JIO= Jejunoileal obstruction, SBO= Small bowel obstruction. TOP= Termination of pregnancy. IUFD = Intrauterine fetal demise.

**Table 3:** Additional information on cases ending in intrauterine fetal death (IUFD)

| Study | IUFD (n) | Definition of isolated vs. non-isolated based on ultrasound findings and/or genetic testing | Level of obstruction – Isolated vs. non-isolated | Gestational age at IUFD | Findings |
| --- | --- | --- | --- | --- | --- |
| Taşdemir (2025) | 1 | Ultrasound findings + genetic testing | DO – non-isolated (1)  *No autopsy* | 37 weeks | Agenesis of septum pellucidum, bilateral radial agenesis, normal karyotype |
| Heinrich (2023) ^9^ | 10 | Ultrasound findings | DO – isolated (5)  DO – non-isolated (5)  *Autopsy in 5/10 cases* | 30+5, 33+3, 33+5, 34+2, 34+6  32+4, 32+4, 33+0, 33+3, 34+0 | -  T21 (2), ventricular septal defect (1), persistent left vena cava superior (1), multicystic dysplastic kidney (1) |
| Zmora (2023) ^44^ | 1 | Ultrasound findings + genetic testing | SBO – isolated (1)  *No autopsy* | 32 weeks | Normal karyotype(1) |
| Mangione (2023) ^41^ | 4 | Ultrasound findings | JIO – isolated (2)  JIO – non-isolated (2)  *No autopsy* | - | -  Chromosomal anomaly 46XY,der(13)t(2;13) (1), other not reported |
| Wu (2022) ^40^ | 2 | Not reported | DO – not reported (1)  JIO – not reported (1)  No details on autopsy | -  - | -  - |
| Bishop (2020) ^28^ | 1 | Genetic testing | DO – non-isolated (1)  *No autopsy* | - | T21 |
| Yin (2020) ^29^ | 1 | Genetic testing | DO – isolated (1)  *Confirmed on autopsy* | 34 weeks | Normal karyotype, other structural malformations not specified (1)* |
| Nakamura (2019) ^8^ | 4 | Not reported | DO – isolated (1)  DO – non-isolated (1)  JIO – isolated (1)  JIO – non-isolated (1)  *Confirmed on autopsy* | 31+3  32+6  36+5  34+2 | -  -  -  - |
| Yang (2018) ^30^ | 2 | Not reported | DO – unknown (2)  *No details on autopsy* | - | - |
| Rubio (2017) ^38^ | 1 | Ultrasound findings + genetic testing | JIO – non-isolated (1)  *Confirmed on autopsy* | 26 weeks | Anal atresia (1) |
| Kim (2016) ^31^ | 2 | Ultrasound findings | DO – isolated (1)  DO – non-isolated (1)  *No details on autopsy* | 37+2 weeks  26+0 weeks | -  Double-outlet right ventricle and ventricular septal defect, no chromosomal analysis |
| Kucinska-Chahwan (2015) ^32^ | 2 | Not reported | Not reported  *No details on autopsy* | - | - |
| Choudhry (2009) ^33^ | 3 | Not reported | Not reported  *Confirmed on autopsy* | - | - |
| Ruiz (2009) ^39^ | 2 | Not reported | Not reported  *No details on autopsy* | - | - |
| Cohen Overbeek (2008) ^34^ | 3 | Ultrasound findings + genetic testing if available | DO – non-isolated (3)  *Autopsy in 2/3 cases* | 33+3, 33+4,  38 weeks | T21 (2), Esophageal atresia with fistel (1) |
| Brantberg (2002) ^7^ | 4 | Ultrasound findings + genetic testing if available | DO – isolated (2)  DO – non-isolated (2)  *Confirmed on autopsy* | 31+2, 33+3 weeks  35+4, 33+6 weeks | -  Hypoplastic gallbladder (1)  Atrial septal defect, coarctation of the aorta, adduction of thumbs, vertebral anomalies, non-lobulation of lungs, Goldenhar syndrome (1) |

Only first author is given for each study. IUFD= Intrauterine fetal demise, DO= Duodenal obstruction, JIO= Jejunoileal obstruction, , SBO= Small bowel obstruction, IO= Ileal obstruction

**Appendix A: Quality in Prognosis studies (QUIPS) tool**

| **Study** | **Study participation** | **Study attrition** | **Prognostic factor measurement** | **Outcome measurement** | **Study confounding** | **Statistical analysis and reporting** | **Overall** |
| --- | --- | --- | --- | --- | --- | --- | --- |
| Taşdemir, 2025 | Low | Low | Low | Low | Low | Low | Low |
| Heinrich, 2023 | Low | Low | Low | Low | Moderate | Low | Low |
| Zmora, 2023 | Moderate | Low | Moderate | Low | High | Low | Moderate |
| Mangione, 2023 | Low | Moderate | Low | Low | High | Low | Moderate |
| Wu, 2022 | Moderate | Moderate | High | Low | High | Low | High |
| Gupta, 2021 | Low | Low | Low | Low | High | Low | Moderate |
| Bishop, 2020 | Low | Low | Low | Low | High | Low | Moderate |
| Yin, 2020 | Moderate | Low | Low | Low | High | Low | Moderate |
| Orgul, 2019 | Low | Low | Low | Moderate | Moderate | Low | Low |
| Nakamura, 2019 | Low | Low | Low | Low | Low | Low | Low |
| Bartholmot, 2019 | Low | Low | Moderate | Low | Low | Low | Low |
| Yang, 2018 | Low | Low | Moderate | Moderate | High | Low | Moderate |
| Rubio, 2017 | Moderate | Low | Moderate | Low | High | Low | Moderate |
| Kim, 2016 | Low | Moderate | Low | Low | Moderate | Low | Low |
| Kucinska-Chahwan, 2015 | Low | Low | Moderate | Moderate | High | Low | Moderate |
| Choudry, 2009 | Low | Low | High | Moderate | High | Low | High |
| Ruiz, 2009 | Low | Low | High | Moderate | High | Low | High |
| Cohen Overbeek, 2008 | Low | Low | Low | Low | Low | Low | Low |
| Garel, 2006 | Moderate | Moderate | Moderate | Low | High | Low | Moderate |
| Brantberg, 2002 | Low | Low | Low | Low | Low | Low | Low |
