# Supplementary material for: Mortality in prenatally detected congenital small bowel obstructions: systematic review and meta-analysis
Source: AJOG Glob Rep. 2026 Feb 23;6(2):100619. doi: 10.1016/j.xagr.2026.100619 (PMC13019568; doi:10.1016/j.xagr.2026.100619)

**Appendix C:** Forest plot illustrating the (pooled) proportions and prediction intervals of intrauterine fetal death (IUFD) prevalence among fetuses with a jejunoileal obstruction


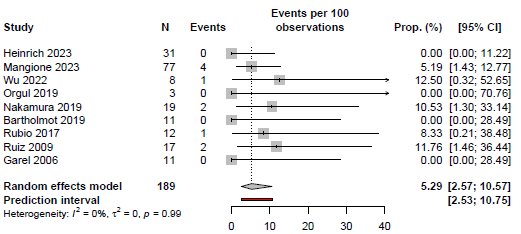

Supplement: Supplementary file 5 [file mmc5.docx]
